# Infestation and Identification of Ixodid Tick in Cattle: The Case of Arbegona District, Southern Ethiopia

**DOI:** 10.1155/2016/9618291

**Published:** 2016-12-26

**Authors:** Jelalu Kemal, Nateneal Tamerat, Temesgen Tuluka

**Affiliations:** ^1^Haramaya University, College of Veterinary Medicine, P.O. Box 138, Dire Dawa, Ethiopia; ^2^Sidama Zone Agricultural and Rural Development Office, Southern Ethiopia, Ethiopia

## Abstract

The study was conducted from October 2014 to June 2015 to estimate tick prevalence and identify major tick genera infesting cattle and the associated risk factors in Arbegona district, southern Ethiopia. A total of 2024 adult ticks were collected from main body parts of animals and eight species of ticks which belong to three genera were identified. Questionnaire survey was employed concerning the general case on the tick infestation problems on the cattle. From 384 cattle examined, 291 (75.7%) were found to be infested with one or more types of tick species. The relative prevalence of each genera was* Amblyomma* (34.9%),* Rhipicephalus (Boophilus)* (26.6%),* Hyalomma* (19.2%), and* Rhipicephalus* (19%). The prevalence of tick infestation in good (65.5%), medium (74%), and poor body condition animal (100%) was found to be statistically significant (*p* < 0.05). There was also significantly (*p* < 0.05) higher prevalence in old (98.4%) than adult (78.8%) and young (59.8%) age groups of animals. In the survey, 87.5% of respondents believe that there was tick infestation problem in their locality. This study showed there was high burden and prevalence of ticks that still play major roles in reducing productivity and cause health problems of cattle in the area which call for urgent attention.

## 1. Introduction

Ethiopia has Africa's largest livestock record with an estimated total cattle population of 57.83 million [1]. Currently, livestock has been contributing to the livelihoods of estimated 80% of the rural human population of the country [2]. The current utilization of hides and skins is estimated to be 48% for cattle which accounts for 12–16% of the total value of exports in the country [3]. However, the contribution from this huge livestock resource to the national income of the country is disproportionately small due to several factors. Ticks are a global problem and considered as a major obstacle in the health and livestock productivity that cause considerable economic losses [2, 4]. A conservative estimate of USD 45,269.35 (1 million ETB) loss annually was made through rejection and downgrading of hides and skins in Ethiopia [5, 6].

According to Walker et al. [7] ticks in Africa with veterinary importance comprise about more than forty species. Among these the most important tick species in Ethiopian cattle's are* Amblyomma, Hyalomma, and Rhipicephalus (Boophilus)* [8]. The country environmental condition and vegetation are highly conducive for ticks and tick-borne disease maintenance [5]. The life of ticks depends on the host animal which results in retardation of animal growth, loss of milk, and meat production, generally affecting the market and decreasing the annual income. Many people who live at rural area depend on the livestock production, which have faced to a considerable economic crisis due to tick infestation of cattle in the study area (source: district agricultural office). Tick infestation has been known to cause a great deal of loss or reduction of productivity by influencing the performance and qualities of the animal yield in the area which in turn leads to reduction of this sector contribution towards the country's development. Acaricide application is still the main method of tick control in Ethiopia [8]. Currently, organophosphates are the most widely used chemicals although evidence of resistance is emerging [9].

Although considerable amount of research has been done regarding ixodid ticks infestation in Ethiopia, it is still relevant to generate periodic and recent information about the prevalence of different species of ticks with the associated factors along different parts of the country. Consequently, minimizing the economic losses from tick infestation different studies in various parts of the country is needed so far. Furthermore, there was no known research conducted in the past and no any published information regarding tick infestation in cattle in the study area. The objectives of this study were aimed to identify and estimate the prevalence of various tick species and assess major factors that could contribute to tick infestation on cattle in the study area.

## 2. Materials and Methods

### 2.1. Description of the Study Area

**Figure 1:**
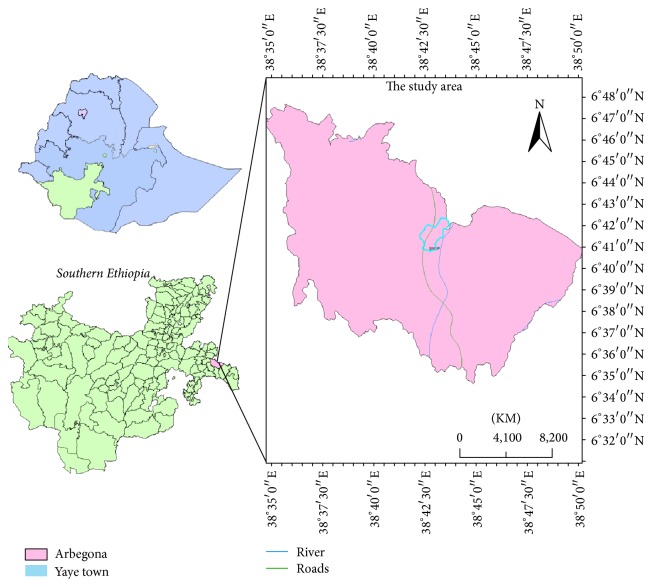
Map representing the study area (Arbegona district).

The study was carried out in Arbegona district which is located at Sidama zone in southern Ethiopia about 344 km far from Addis Ababa, capital city of Ethiopia (Figure 1). Geographically, the district is located at 6°41′N latitude and 38°43′E longitude with an elevation of 8486 ft. Arbegona is bordered on the north by the Oromia Region, on the south by Bona Zuria, on the northwest by Gorche, on the southwest by Bursa, and on the east by Bensa. According to a 2004 central statistics agency report, Arbegona had 36 kilometers of all-weather roads and 25 kilometers of dry-weather roads. Climatically, Arbegona district belongs to the southern Ethiopia high land and it is mainly characterized by two agro ecological zones. These are mid-land (10%) and arable high land (90%). The district has 36 rural and 3 urban Kebeles (peasant associations (PA's) that usually consists of up to 200 households). Its altitude ranges from 2000 to 3300 meters above sea level (m.a.s.l.) with average rainfall ranging from 1600 to 1900 millimeter and the annual temperature varies between 7°C and 21°C [10]. Cattle are main assets and savings of the people and important source of protein and energy in their diet. Cattle by-products in rural areas mainly comes from indigenous zebu breed kept in traditional management system. Currently, cross breed cattle are increasing in number that primarily concentrate around urban and periurban areas where farmers supply their by-products to urban consumers. In this district free grazing has been practiced over many generations predominantly located at valley bottoms or on wet lands favorable for tick infestation. The land of Arbegona is comparatively moist than the nearby districts of Sidama zone which has lowest cultivated crop coverage (100 hectare) since most of the land of this district is more than 2400 m.a.s.l. in the Moist-dega and in Moist-wurch. It has also large open grazing land coverage. The land in the district is well covered with grass and vegetation and also reports show that some 4080 ha of the land is covered with bamboo forests.

### 2.2. Study Population

The study animals were cattle of all age, sex, breed, and body condition scores found in the three selected Kebeles of Arbegona district. The animals depend on grazing throughout the year for their feed sources with little supplementation of crop residues.

### 2.3. Study Design

A cross sectional study was conducted from October, 2014, to June, 2015, to estimate the prevalence of ticks, identification of the major ticks genera and species, their predilection sites, and burden along different age groups, breeds, season, body conditions, sex of animals, and different areas in the district. All the animals selected as sampling unit were checked for any tick infestation based on the number of ticks found on the animal and the study record period. Ticks were collected from ears, heads, dewlaps, belly/flunk, udder/scrotum, fore/hind legs, perineum, and tails in the separated sample bottles with 70% ethyl alcohol (ethanol).

### 2.4. Method and Sources of Data Collection

The data for this study were collected from primary and secondary data sources. Questionnaires, interview, and observation as well as focus group discussion were used as primary sources to collect data from respondents concerning to tick infestation problems on the cattle's from their local area. All questionnaires were arranged in sequential manner that helps discussion and analysis. During the study period, 40 model farmers and animal health assistants from both sexes were considered for questionnaire survey to give their responses on major factors that lead cattle to tick infestation problem and 3 Kebeles were selected purposively according to accessibility and cattle population, proximity to livestock market, and other socioeconomic characteristics of the areas. Simple random sampling method was employed to select the study individual animals. The secondary data were collected by referring the district recorded document such as unpublished book and check list from the district agricultural office like case books concerning the general case on the tick infestation problem on the cattle. The age of the cattle was grouped into young (1 to 2 years), adult (3 to 7 years), and old (>8 years) according to Gatenby [11] and Abera et al. [4], while body condition score was employed after categorizing the animals into poor, medium, and good according to Nicholson and Butterworth [12] after some modification. Extremely lean cattle, having prominent dorsal spines pointed to the touch and individual visible transverse processes into which a finger could be easily pushed, were considered as poor body condition score. A medium body condition score cattle was expressed as having usually visible ribs with little fat cover and barely visible dorsal spines. A good body condition score was given for the animals when fat cover easily seen in critical areas and felt and the transverse processes were not seen or felt. During the study, samples were collected in both dry and wet season of the year.

### 2.5. Sample Size Determination

The sample size was determined by using the formula given in Thrusfield [13]. Accordingly, with considering the expected prevalence of 50% and 5% absolute precision with 95% confidence interval, a total of 384 cattle were included in the study.

### 2.6. Tick Collection, Identification, and Count

The entire body surface of the animal was examined thoroughly for the presence of any tick and all visible adult ticks were collected from half-body on alternative sides. Ticks were removed carefully and gently in a horizontal pull to the body surface. The collected ticks were preserved in universal bottles containing 70% ethyl alcohol and labeled with the animal identification and predication site, age, sex, and data of collection. The specimens were transported to the parasitology laboratory of the school of veterinary medicine of Hawassa University for counting and identification. Ticks were counted and subsequently identified to genus and species level by using stereomicroscope, according to standard identification keys given by Walker et al. [7]. The half-body tick counts of cattle were doubled to obtain the whole body tick burdens. During examination of the selected animals for tick infestation, the age, sex, body condition score, breed, and Kebele of the sampled animals were recorded on a special format designed for this purpose. During the study, distribution of ticks and total count of each tick genera were done.

### 2.7. Data Analysis

The data were entered and managed in Microsoft-excel sheet. SPSS 20.0 version software program was employed for the data analysis. The overall prevalence of tick was determined by dividing the number of positive animals by total sample size and was expressed as percentage. Chi-square (*χ*^2^) test was used to assess the association in tick infestation between different variables. Effects were reported as statistically significant in all cases if *p* value is less than 5% (*p* < 0.05) and multivariable logistic regression was used to see the association of risk factors.

## 3. Results

### 3.1. Tick Infestation in Cattle and Different Risk Factors

Out of the total 384 cattle examined for the presence of ticks, 291 (75.5%) were found to be infested with varying numbers of tick genera (Table 1). Higher tick prevalence was recorded in Charicho Kebele (85%) and slightly lower prevalence in Gute (68.7%) with no statistically significant difference (*p* > 0.05). The occurrence of tick infestation in sex of animals was also not significantly different (*p* > 0.05). Tick infestation of animals with age and different body conditions showed that there were statistically significant variations (*p* < 0.05). During this study, there was higher prevalence of tick infestation in wet season (77.6%) than dry season of the year (72.7%) (Table 1).

### 3.2. Tick Burden and Species Identification

**Figure 2:**
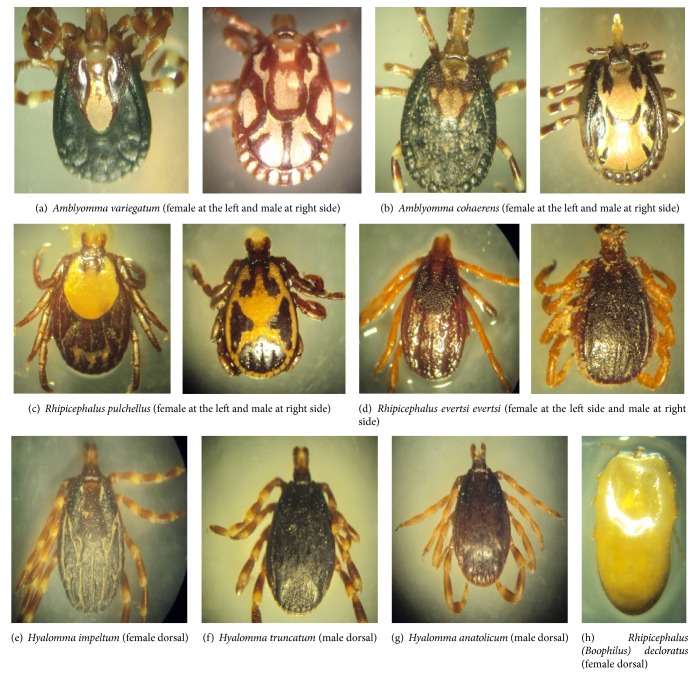
Representative pictures of identified different tick species of cattle during the study in the area.

A total of 2024 adult ticks were collected from 291 cattle in the study sites (Table 2). Eight different tick species were registered from three genera including* Boophilus* subgenus of* Rhipicephalus* during the study period: two* Amblyomma* species (Figures 2(a) and 2(b)), two* Rhipicephalus* (Figures 2(c) and 2(d)), three* Hyalomma* (Figures 2(e), 2(f), and 2(g)), and* Rhipicephalus (Boophilus) decloratus *(Figure 2(h)).

### 3.3. Distribution of Tick Species on Body Parts of Study Animals

*Amblyomma* and* Rhipicephalus (Boophilus)* appeared to be dominant on the dewlap region of the animal while* Hyalomma* ticks prefer the sternum area next to dewlap of the animal. On the other hands,* Rhipicephalus* ticks tend to attach in the anal and tail section followed by ear area of the animal (Table 3).

### 3.4. Different Model Farmers and Animal Health Assistant Questionnaires

Over 87.5% of the respondents acknowledged tick infestation of animal as problem while 35% of them know the presence of tick-borne diseases. Among the participant farmers, 65% of them recognize the effort of local veterinary workers toward minimizing and control of tick infestation while 55% of them believe that the beginning of rainy season favors tick infestation (Table 4).

## 4. Discussion

In the present study high overall prevalence of ticks (75.7%) was registered (Table 1). Similarly, high prevalence of ixodid ticks was reported from different part of the country including 82% [14], 81.25% [15], 74% [16], and 65.5% [17]. The high overall prevalence of tick infestation in cattle was also recorded by other authors such as Regassa [8] and Ayalew et al. [18] in the eastern and central part of Oromia, respectively. Similarly, higher finding was reported by de Castro [19] where it was stated that more than 80% of the cattle studied were ticks-infested. Abera et al. [4] reported around 95% tick infestation prevalence in south western Ethiopia. Our study is not in line with the finding reported by Tiki and Addis [20] with a prevalence of 25.64%. The inconsistency among these studies could be attributed to a wide range of factors including agroecological, animal health practice, or management difference with in their respective study areas. In this particular study, there is no significant difference (*p* > 0.05) of tick infestation within three Kebeles of the district (Table 1). This is probably due to similarities in agroecological setting and animal health practice in these study sites.

In this study,* Amblyomma* was found to be the most abundant tick genera which accounts for 34.9% of the total finding. Likewise, Pawlos and Derese [21] indicated* Amblyomma *as the leading tick genera with 43.46% prevalence. This finding is also in agreement with that of previous reports on a high number of* Amblyomma* in three agroecological zones in central Oromia by Ayalew et al. [18] and at Haramaya University by Yehualashet et al. [22].* Amblyomma variegatum* and* Amblyomma cohearens* were the two species of the genera identified during the study period in the area. Such finding echoes the alarming need of intervention since* Amblyomma* ticks are a potential vector for a disease caused by* Cowdria rumintium* [23] which is common where this tick is prevalent in the country.* Rhipicephalus (Boophilus)* was the second most abundant tick subgenus (26.6%) in this study. Mekonnen et al. [23] described* Rhipicephalus (Boophilus)* as the commonest and most wide spread tick in Ethiopia. Similar to the current finding, Abebe et al. [24] reported* Rhipicephalus (Boophilus) decoloratus* (24.83%) as the second abundant tick species while Tessema and Gashaw [25] indicated 15.4% prevalence. On the contrary, the findings of Alekaw [26] at Metekel Ranch, Ethiopia, showed a lower prevalence (5.7%) of* Rhipicephalus (Boophilus) *tick species. This may be due to the geographical location and altitude factors which belongs to lower area of the country with 1500 to 1600 m.a.s.l. of Metekel ranch. The identified tick species of this genera were* Rhipicephalus pulchellus, Rhipicephalus evertsi evertsi, *and* Rhipicephalus (Boophilus) decloratus.* Female* Rhipicephalus (Boophilus)* were abundant from September to April and could transmit* Babesia bigemina*, in addition to anorexia and anemia in case of severe infestation [27]. Season-wise, Shiferaw [28] indicated that* Rhipicephalus (Boophilus)* had highest frequency during dry seasons (January, February, and early March) in the observed area of Wolaita Zone.

*Hyalomma* is the third most abundant tick genera (19.2%) in this study which is in compatible with Tessema and Gashaw [25]. Likewise, Getachew et al. [15] reported* Hyalomma *as the second most abundant tick with 20.34% prevalence. Lower prevalence (5%) of* H. m. rufipes* was reported by Ayalew et al. [18] from Sebeta Waso District. In the current study, the fourth and least abundant tick genera were* Rhipicephalus* having a prevalence of 19%. This finding was close to the works conducted by Mekonnen et al. [23] at Ghibe Tullary in central Ethiopia who reported 21.2% prevalence. The finding is also consistent with Ayalew et al. [18] who found 19.5%. This tick genus shows no apparent preference for any particular altitude, rainfall, or season [5].* Hyalomma impeltum, Hyalomma truncatum, *and* Hyalomma anatolicum* were tick species of this genera identified in the study area which are possible and potential vectors for* Babesia*,* Rickettsia,* and* Theleria* diseases [29].

With regard to distribution pattern and predilection site of ticks,* Amblyomma *and* Boophilus* had relatively fair distribution to almost all the examined body regions of animals.* Rhipicephalus* ticks were restricted to the anal region and under tail and ear areas, with very few of them observed on the scrotal, udder, dewlap, and sternum area which is also true in the work of Nateneal et al. [14] who reported finding of* Rhipicephalus evertsi evertsi *exclusively in perineum and anal area.

In the present study, among the considered variables as a factor for tick prevalence, only age and body condition groups had significant association (*p* < 0.05) with prevalence of tick. Tick burden was significantly (*p* < 0.05) higher in older animals than the other age groups. This is probably associated with low immunity and resistance of old animals. Regarding body condition, animals with poor body condition showed significantly (*p* < 0.05) higher tick infestation than the other groups. This may be due to the fact that poorly conditioned animals had low resistant to tick infestation and lack enough body capacity to build resistance whereas animals with good body condition showed reasonable combat to the infestation according to [30]. On the way, tick infestation might be a cause for poor body condition instead of vice versa.

In the current study, there was no considerable difference (*p* > 0.05) in the prevalence of ticks within the wet and dry season. However, Mohamed et al. [31] report indicates significantly (*p* < 0.05) increased prevalence of tick in wet season than dry season. Mekonnen et al. [23] reported that ticks were found on cattle throughout the study period, although higher tick counts were observed during the rainy than dry season. The most important environmental factors that influence the occurrence of ticks in a biotope include climate such as temperature and relative humidity [32]. Even if the same factor affects the survival of all tick species to varying degrees, each tick species has its particular threshold temperature and moisture during their life time. The survival of ticks also depends on the presence of hosts suitable for reproduction by the adults [7]. For instance,* Rhipicephalus (Boophilus)* species are adapted to feed on cattle, but some may survive by feeding on sheep or antelope.

The present questionnaire survey result revealed that the entire respondents' know or had information about the ticks. 87.5% of the interviewed participant believe that there was tick infestation problems in livestock in their locality. Ticks affect livestock in general and cattle in particular by reducing milk production, growth, hide and skin, and birth rate. From the total respondents interviewed, 60% confirmed that hard ticks are more common and affect livestock productivity in their locality. The same percentage of respondents also identified hard tick with that of soft ticks infesting cattle. According to the respondents analysis, tick infestation occurs throughout the year, but majority of them described tick infestation was most favored at the beginning of rain season followed by the dry and mid rain season with lower prevalence at the end of rain season. Comparable findings were recorded in Jimma Zone [33] and in Borena pastoral area [34, 35]. The questionnaire survey finding indicated only 35% of the participants know tick-borne diseases transmitted by ticks while the remaining 65% revealed that they do not know tick-borne diseases. This showed effects and constraints of ticks in livestock productivity and health impact has been not well understood by the community in the area. The present study also revealed that cross breed cattle are comparatively more susceptible to tick infestation compared to indigenous breed cattle in their locality. Similar result was also reported by other researchers in the country with high susceptibility of cross or exotic breed cattle than local breeds to tick infestation [36]. In the present questionnaire survey, 65% of the respondents acknowledge the involvement of district veterinary workers in the control of tick infestation even though there is no well-planned program of tick control strategy in the study site in particular and the country in general except on dairy farms [37].

## 5. Conclusion

The study demonstrated that there was high burden of ticks in the area with overall prevalence of 75.5% which indicates ticks are common and important ectoparasite of cattle in Arbegona district. This study showed that there was high burden and prevalence of ticks that still play major roles in reducing productivity and cause health problems of cattle in the area. Further detailed studies on the role of different ticks species in causing disease in cattle and their economic consequence to the livelihoods call for urgent attention.

## Figures and Tables

**Table 1:** Potential risk factors for tick infestation status of cattle in Arbegona district.

Risk factors	Number of animals examined	Number of positive animals	95% CI	*χ* ^2^	*p* value
*Kebeles*					
Charicho	128	109 (85%)	—	1.402	0.705
Gute	128	88 (68.7%)	47.4–68.5		
Yaye 01	128	94 (73.4%)	53.8–73.4		
*Sex*					
Female	249	290 (76.3%)	—	0.559	0.454
Male	135	101 (74.8%)	55.7–75.8		
*Age*					
Old	63	62 (98.4%)	—	6.154	0.046
Adult	194	153 (78.8%)	56.7–68.0		
Young	127	76 (59.8%)	42.4–68.8		
*Body condition*					
Poor	32	32 (100%)	—	6.812	0.000
Medium	306	227 (74%)	46.6–58.9		
Good	46	32 (69.5%)	69.1–100		
*Season*					
Wet	237	184 (77.6%)	—	1.023	0.312
Dry	147	107 (72.7%)	51.1–67.9		

*Total*	*384*	*291 (75.7%)*			

**Table 2:** Proportion of ticks identified in Arbegona district.

Tick genera	Proportion
*Amblyomma*	708 (34.9%)
*Rhipicephalus (Boophilus)*	540 (26.6%)
*Hyalomma*	389 (19.2%)
*Rhipicephalus*	387 (19%)

*Total*	*2024 (100%)*

**Table 3:** Genera of ticks and their distribution on body regions of cattle in Arbegona district.

Body region	*Amblyomma*	*Rhipicephalus (Boophilus)*	*Hyalomma*	*Rhipicephalus*	Total
	+ve (counted)	+ve (counted)	+ve (counted)	+ve (counted)	+ve (counted)
Dewlap	31 (234)	26 (174)	19 (163)	2 (7)	78 (578)
Udder	28 (226)	17 (114)	9 (35)	3 (9)	57 (384)
Scrotum	22 (157)	—	6 (31)	2 (8)	30 (196)
Anal region, under, tail	—	20 (131)	7 (14)	25 (194)	52 (339)
Sternum	18 (91)	19 (121)	15 (146)	3 (11)	55 (369)
Ear	—	—	—	19 (158)	19 (158)

*Total*	*99 (708)*	*82 (540)*	*56 (389)*	*54 (387)*	*291 (2024)*

**Table 4:** Questionnaire data representing the question items and respondents response.

Question contents	Alternatives	Respondents		
		Male	Female	Total (%)
Know the ticks	Yes	28	12	40 (100)
	No	—	—	— (0)

Tick problems in your locality	Yes	25	10	35 (87.5)
	No	3	2	5 (12.5)

Type of ticks you know in your locality	Hard tick	16	8	24 (60)
	Soft tick	4	2	6 (15)
	Both	8	2	10 (25)

Type of ticks that seriously damage (affect) cattle in your local area	Hard tick	18	6	24 (60)
	Soft tick	8	2	10 (25)
	Both	4	2	6 (15)

Know the season of tick infestation outbreak	Yes	28	12	40 (100)
	No	—	—	— (0)

Season of tick infestation outbreak	At the end of rain season	2	1	3 (7.5)
	At the beginning of rainy season	16	6	22 (55)
	In mid of rainy season	4	3	7 (17.5)
	At the dry season	6	2	8 (20)

Know any tick-borne disease	Yes	10	4	14 (35)
	No	18	8	26 (65)

Species of livestock mostly infected by ticks	Bovine	28	12	40 (100)
	Ovine, caprine, others	—	—	—

Breed of cattle comparatively more susceptible to the tick infestation in your locality	Indigenous breed	9	8	17 (42.5)
	Cross breed	19	4	23 (57.5)

District veterinary workers contribute to minimize and control the prevalence of tick and tick infestation	Yes	18	8	26 (65)
	No	10	4	14 (35)
